# Analysis of single nucleotide polymorphisms of the metabotropic glutamate receptors in a transgender population

**DOI:** 10.3389/fendo.2024.1382861

**Published:** 2024-06-11

**Authors:** Rosa Fernández, Karla Ramírez, Roberto Lorente-Bermúdez, Esther Gómez-Gil, Mireia Mora, Antonio Guillamon, Eduardo Pásaro

**Affiliations:** ^1^ Department of Psychology, Interdisciplinary Center for Chemistry and Biology Institute, Centro Interdisciplinar de Química e Bioloxía (CICA), Diagnóstico Conductual y Molecular Aplicado a la Salud (DICOMOSA) Group, University of A Coruña, A Coruña, Spain; ^2^ Department of Psychology, Institute for Biomedical Research of A Coruña (INIBIC), A Coruña, Spain; ^3^ Gender Identity Unit, Psychiatry Service, Institute of Neurosciences, Hospital Clínic Institut d’Investigacions Biomèdiques August Pi i Sunyer (IDIBAPS), Barcelona, Spain; ^4^ Department of Endocrinology and Nutrition, Hospital Clínic Institut d’Investigacions Biomèdiques August Pi i Sunyer (IDIBAPS), Barcelona, Spain; ^5^ Department of Psychobiology, Faculty of Psychology, National University of Distance Education (UNED), Madrid, Spain

**Keywords:** gender dysphoria (GD), gender incongruence, membrane-bound estrogen receptors, mERs, metabotropic glutamate receptors (mGluR), mGluR5, MGluR7, rapid estradiol signaling

## Abstract

**Introduction:**

Gender incongruence (GI) is characterized by a marked incongruence between an individual’s experienced/expressed gender and the assigned sex at birth. It includes strong displeasure about his or her sexual anatomy and secondary sex characteristics. In some people, this condition produces a strong distress with anxiety and depression named gender dysphoria (GD). This condition appears to be associated with genetic, epigenetics, hormonal as well as social factors. Given that L-glutamate is the major excitatory neurotransmitter in the central nervous system, also associated with male sexual behavior as well as depression, we aimed to determine whether metabotropic glutamate receptors are involved in GD.

**Methods:**

We analyzed 74 single nucleotide polymorphisms located at the metabotropic glutamate receptors (mGluR1, mGluR3, mGluR4, mGluR5, mGluR7 and mGluR8) in 94 transgender *versus* 94 cisgender people. The allele and genotype frequencies were analyzed by c2 test contrasting male and female cisgender and transgender populations. The strength of the associations was measured by binary logistic regression, estimating the odds ratio (OR) for each genotype. Measurement of linkage disequilibrium, and subsequent measurement of haplotype frequencies were also performed considering three levels of significance: P ≤ 0.05, P ≤ 0.005 and P ≤ 0.0005. Furthermore, false positives were controlled with the Bonferroni correction (P ≤ 0.05/74 = 0.00067).

**Results:**

After analysis of allele and genotypic frequencies, we found twenty-five polymorphisms with significant differences at level P ≤ 0.05, five at P ≤ 0.005 and two at P ≤ 0.0005. Furthermore, the only two polymorphisms (rs9838094 and rs1818033) that passed the Bonferroni correction were both related to the metabotropic glutamate receptor 7 (mGluR7) and showed significant differences for multiple patterns of inheritance. Moreover, the haplotype T/G [OR=0.34 (0.19–0.62); P<0.0004] had a lower representation in the transgender population than in the cisgender population, with no evidence of sex cross-interaction.

**Conclusion:**

We provide genetic evidence that the mGluR7, and therefore glutamatergic neurotransmission, may be involved in GI and GD.

## 1 Introduction

Gender identity is the consciousness of being a man or a woman. For the majority of the population this awareness is congruent with the male or female aspect of their genitals; they are cisgender men or women (CM and CW respectively). However, for some people, gender identity is not congruent with their genitalia (ICD-11; 1), 2022), they are transgender men or women (TM and TW respectively). The transgender population is a heterogeneous group: some feel they belong to the binary male-female dichotomy (binary transgender people), while others feel they are agender, bigender, gender fluid, etc., they are non-binary transgender people (2). In some transgender persons the gender incongruence could drive deep depression and anxiety, called gender dysphoria (GD) by DSM-5 (3), that require gender affirming hormone treatment (4).

Since the studies of Swaab (5) on post mortem brains of male and female cisgender and female transgender people showing that the central part of the bed nucleus of the stria terminalis was feminized in female transgender, genetic and neuroimaging *in vivo* studies have been carried out within the framework of sexual differentiation, and a cortical neurodevelopmental theory has been put forward (6, 7). The genetic analysis of sex steroid receptor polymorphisms (8–16) and coactivator polymorphisms (17) in transgender population support the above mentioned theory.

Within the scope of sex differences, polymorphisms of the α and β estrogen receptors as well as of the androgen receptor present particular interest (13). In transgender women, complex interaction between estrogen and androgen receptor polymorphisms was unveiled. Thus, an inverse allele interaction between rs113770630 (ERβ) and rs193922933 (AR) is characteristic of the TW population: when either of these polymorphisms is short, the other is long. rs9340799 (ERα) and rs113770630 (ERβ) are also related to the TM population although no interaction between these two polymorphisms was evidenced (13).

The ERα and ERβ receptors were initially identified as intracellular, ligand-regulated transcription factors that are expressed throughout the body, and in numerous brain regions (18). But this classical mechanism only partially explains the wide variety of effects produced by estradiol (19). Thus, recent studies suggest that the rapid effects of estrogens are the result of a novel signaling mechanism from the cell membrane (20) resulting from the coupling of classical ERs localized at the membrane (mERs) with metabotropic glutamate receptors (mGluRs) (21). In the central nervous system, the signaling cascades initiated by the mER/mGluR coupling has been shown to be involved in many physiological functions in both sexes (19).

The mGluRs are a heterogeneous group that is categorized into three subgroups based on sequence homology, pharmacology, and downstream signaling (22): Group I, consists of mGluR1 and mGluR5, group II includes mGluR2 and mGluR3, while receptors mGluR4, mGluR6, mGluR7, mGluR8 form group III. Evidence of ER interactions with the three mGluR groups has been found. Group I is primarily characterized by their postsynaptic localization and their association with Gq G proteins. Group II is primarily localized presynaptically and associate with Gi/o G proteins. While Group III is also primarily expressed presynaptically and couple to Gi/o G proteins (23). All these receptors are expressed in the brain (23).

At behavioral level, mGluRs are implicated in sexual behavior. Specifically, mGluR5 and mGluR7 receptors influence rodent male sexual behavior (24–26). Furthermore, Schwarz and McCarthy reported that antagonizing glutamate receptors during the critical period of sexual differentiation blocks estradiol-induced defeminization but not masculinization of behavior in adulthood (27).

Moreover, mGluR receptors are of particular interest for transgender people because they show higher rates of attempted and suicidal ideation compared to the overall population (28, 29) and they are nearly twice as likely to die than cisgender people (30). Interestingly, it is worth noting that glutamatergic transmission is dysregulated in suicidal individuals (28, 31, 32).

Because of the coupling of classical ERs and mGluRs, and the implications of both types of receptors in brain sex differences and behavior, in order to delve into the molecular bases of gender identity, we analyzed 74 polymorphisms located at the metabotropic glutamate receptors (mGluR1, mGluR3, mGluR4, mGluR5, mGluR7 and mGluR8) in a homogeneous male and female transgender *versus* to a male and female cisgender populations.

## 2 Methods and materials

### 2.1 Participants

The analyzed population consisted of 47 transgender women (TW) and 47 transgender men (TM) diagnosed and recruited through the Gender Identity Unit of the Clínic Hospital of Barcelona (Spain), and 50 cisgender women (CW) and 44 cisgender men (CM) obtained from the biobank of the Regional University Hospital of Málaga (Spain).

The inclusion criterion for the entire population that participated in the study was being older than 18 years, and specifically for the transgender population, the presence of the first symptoms of GI (ICD-11) before puberty (defined as early onset).

The exclusion criteria for all participants were: presence of medical or psychiatric disorders, and previous history of alcohol and/or drug abuse. All participants were matched by geographical origin (Spanish), ethnicity (Caucasian) and the sex assigned at birth. (**Supplementary Table 1**).

### 2.2 Molecular analysis

In the case of the transgender population, genomic DNA was obtained from blood using the DNeasy Blood & Tissue Kit from Qiagen (Madrid, Spain). For the cisgender population, we obtained the DNA samples from the biobank of the Regional University Hospital of Málaga (Spain).

All analyzed polymorphisms were single nucleotide polymorphisms (SNPs) located, according to the Ensembl database (www.ensembl.org/), at the metabotropic glutamate receptors (**Supplementary Table 2**). Genotyping was performed by the microarray Axiom Spanish Biobank (Affymetrix). Statistical analyses were performed using the free online software SNPStats (https://www.snpstats.net/) (33). SNPStats is a simple, free, ready-to-use software which has been designed to analyze genetic-epidemiology studies of association using SNPs. Once the genotype frequencies were uploaded, and for each selected SNP, the following were calculated: allele and genotype frequencies, test for Hardy-Weinberg equilibrium, analysis of association with a response variable based on linear or logistic regression, multiple inheritance models, linkage disequilibrium statistics, haplotype frequency estimation, analysis of association of haplotypes with the response and analysis of interactions (haplotypes-covariate).

The study was approved by the ethical committees of the National University of Distance Education (UNED, Madrid). At the start of the study, written informed consent was obtained from all participants.

### 2.3 Statistical analyses

The genetic analyses were conducted contrasting populations by their sex assigned at birth, considering three levels of significance: *P ≤* 0.05, *P ≤* 0.005 and *P ≤* 0.0005. Moreover, we applied the Bonferroni correction (*P ≤* 0.05/74 = 0.00067) to control the overall type I error rate. The allele and genotype frequencies were analyzed by χ2 test. The strength of the associations with GI was measured by binary logistic regression, estimating the odds ratio (OR) for each genotype for multiple patterns of inheritance. Furthermore, measurement of linkage disequilibrium, and subsequent measurement of haplotype frequencies were performed using logistic regression models to determine the strength of the associations.

## 3 Results

We analyzed the allele and genotypic frequencies, the interactions with the covariate sex assigned at birth, and the linkage disequilibrium of 74 polymorphisms at the metabotropic glutamate receptors (mGluR) (**Supplementary Table 2**), in a transgender population *versus* a cisgender population with similar geographic origin (Spanish) and ethnicity (Caucasian).

The analyses were conducted considering three levels of significance: *P ≤* 0.05, *P ≤* 0.005 and *P ≤* 0.0005, finding significant differences at the three levels: at the *P ≤* 0.05 level, we found significant differences in 25 polymorphisms distributed among the mGluR1, mGluR4, mGluR5, mGluR7, and mGluR8 receptors (**Supplementary Table 2**). At the *P*<0.005 level, five polymorphisms (**Supplementary Table 2**), distributed among the metabotropic receptors mGluR5 and mGluR7, reached statistical significance (rs62237207, rs62237212, rs62237216, rs62237226, rs7782149). While at the *P*<0.0005 level, only two polymorphisms located at the mGluR7, rs9838094 and rs1818033, reached statistical significance. These two polymorphisms that passed the Bonferroni correction, were surrounded by other polymorphisms that reached significance at 0.05 or 0.005 levels (**Supplementary Table 2**).

The analysis of the allele and genotype frequencies with respect to the polymorphism rs9838094, showed significant differences at level *P ≤* 0.0005 and *P ≤* 0.005 respectively (**Table 1**). The ancestral allele G and the genotype G/G were over represented in the transgender population, while genotypes T/G and T/T were more frequent in the cisgender population. The genotype T/T was absent in the transgender population (**Table 1**).

**Table 1:** Analysis of the allele and genotype frequencies for polymorphisms rs9838094 and rs9838094 in trans and cis populations.

rs9838094							
Allele frequencies (n=187)							
	All subjects		Cis group		Trans group		*P*-value
Allele	Count	Proportion	Count	Proportion	Count	Proportion	
G	308	0.82	142	0.76	166	0.89	0.0005†
T	66	0.18	46	0.24	20	0.11	
Genotype frequencies (n=188)							
	All subjects		Cis group		Trans group		*P*-value
Genotypes	Count	Proportion	Count	Proportion	Count	Proportion	
G/G	127	0.68	54	0.57	73	0.78	0.002**
G/T	54	0.29	34	0.36	20	0.22	
T/T	6	0.03	6	0.06	0	0	
NA	1	—	0	—	1	—	
rs1818033							
Allele frequencies (n=188)							
	All subjects		Cis group		Trans group		*P*-value
Allele	Count	Proportion	Count	Proportion	Count	Proportion	
C	259	0.69	113	0.6	146	0.78	0.0003†
G	117	0.31	75	0.4	42	0.22	
Genotype frequencies (n=188)							
	All subjects		Cis group		Trans group		*P*-value
Genotypes	Count	Proportion	Count	Proportion	Count	Proportion	
C/C	89	0.47	34	0.36	55	0.59	0.0009**
C/G	81	0.43	45	0.48	36	0.38	
G/G	18	0.1	15	0.16	3	0.03	

** Reached significance at level *P*≤0.005.

† Reached significance at level *P*≤0.0005.

The analysis of the genotype frequencies according to the different models of inheritance (**Table 2**) showed significant differences for all the inheritance patterns. The analysis of the covariate sex assigned at birth showed no statistical differences between males and females (**Table 3**).

**Table 2:** rs9838094 and rs1818033 polymorphism association analysis with gender incongruence in trans and cis populations, in different models of inheritance.

rs9838094 association with GI (n=187, adjusted by sex)							
Model	Genotypes	Cis group Count/Proportion	Trans group Count/Proportion	OR (95% CI)	*P*-value	AIC	BIC
Codominant	G/G	54 (57.5%)	73 (78.5%)	**Reference 1.00**	5.00E-04*†	252.1	265
	T/G	34 (36.2%)	20 (21.5%)	0.43 (0.23–0.84)			
	T/T	6 (6.4%)	0 (0%)	0.00 (0.00-NA)			
Dominant	G/G	54 (57.5%)	73 (78.5%)	**Reference 1.00**	0.0018*	255.4	265.1
	T/G-T/T	40 (42.5%)	20 (21.5%)	0.37 (0.19–0.70)			
Recessive	G/G-T/G	88 (93.6%)	93 (100%)	**Reference 1.00**	0.0033*	256.5	266.2
	T/T	6 (6.4%)	0 (0%)	0.00 (0.00-NA)			
Overdominant	G/G-T/T	60 (63.8%)	73 (78.5%)	**Reference 1.00**	0.026*	260.2	269.9
	T/G	34 (36.2%)	20 (21.5%)	0.48 (0.25–0.93)			
Log-additive	—	—	—	0.36 (0.20–0.65)	4.00E-04*†	252.5	262.2
rs1818033 association with GI (n=188, adjusted by sex)							
Codominant	C/C	34 (36.2%)	55 (58.5%)	**Reference 1.00**	6.00E-04*†	253.5	266.4
	C/G	45 (47.9%)	36 (38.3%)	0.50 (0.27–0.92)			
	G/G	15 (16%)	3 (3.2%)	0.12 (0.03–0.45)			
Dominant	C/C	34 (36.2%)	55 (58.5%)	**Reference 1.00**	0.002*	256.9	266.6
	C/G-G/G	60 (63.8%)	39 (41.5%)	0.40 (0.22–0.72)			
Recessive	C/C-C/G	79 (84%)	91 (96.8%)	**Reference 1.00**	0.0017*	256.5	266.3
	G/G	15 (16%)	3 (3.2%)	0.17 (0.05–0.60)			
Overdominant	C/C-G/G	49 (52.1%)	58 (61.7%)	**Reference 1.00**	0.19	264.7	274.4
	C/G	45 (47.9%)	36 (38.3%)	0.68 (0.38–1.21)			
Log-additive	—	—	—	0.42 (0.26–0.67)	2.00E-04*†	252.3	262

* Reached statistical significance.

† Passed the Bonferroni correction.

**Table 3:** Interaction analysis of the rs9838094 polymorphism with the covariate natal sex.

Interaction analysis with covariate natal sex								
rs9838094 sex cross-classification interaction (n=187, crude analysis)								
Females					Males			
Genotypes	Cis group	Trans group	OR (95% CI)	*P*-value	Cis group	Trans group	OR (95% CI)	*P*-value
G/G	30	37	**Reference 1.00**	–	24	36	1.22 (0.60–2.46)	0.592
T/G	18	10	0.45 (0.18–1.12)	0.086	16	10	0.51 (0.20–1.28)	0.155
T/T	2	0	0.00	–	4	0	0.00	–
Natal sex within rs9838094 (n=187, crude analysis)								
Genotypes	Natal sex	Cis group	Trans group		OR (95% CI)	*P*-value		
G/G	Females	30	37		**Reference 1.00**	–		
	Males	24	36		1.22 (0.60–2.46)	0.592		
T/G	Females	18	10		**Reference 1.00**	–		
	Males	16	10		1.12 (0.37–3.40)	0.851		
T/T	Females	2	0		**Reference 1.00**	–		
	Males	4	0		1.00	–		
Test for interaction in the trend: 0.76								
rs9838094 within natal sex (n=187, crude analysis)								
Natal sex	Genotypes	Cis group	Trans group		OR (95% CI)	*P*-value		
Females	G/G	30	37		**Reference 1.00**	–		
	T/G	18	10		0.45 (0.18–1.12)	0.086		
	T/T	2	0		0.00	–		
Males	G/G	24	36		**Reference 1.00**	–		
	T/G	16	10		0.42 (0.16–1.07)	0.073		
	T/T	4	0		0.00	–		
Test for interaction in the trend: 0.99								

With respect to the polymorphism rs1818033, the C allele and the C/C genotype were overrepresented in the transgender population (**Table 1**). The association analysis with GI showed significant differences for multiple patterns of inheritance (codominant, dominant, recessive and log-additive) (**Table 2**). The analysis of the covariate sex assigned at birth showed no evidence of sex cross-interaction (**Table 4**).

**Table 4:** Interaction analysis of the rs1818033 polymorphism with the covariate natal sex.

Interaction analysis with covariate natal sex									
rs1818033 sex cross-classification interaction (n=187, crude analysis)									
Females						Males			
Genotypes	Cis group	Trans group	OR (95% CI)		*P*-value	Cis group	Trans group	OR (95% CI)	*P*-value
C/C	18	28	**Reference 1.00**		–	16	27	1.08 (0.46–2.55)	0.870
C/G	26	18	0.45 (0.19–1.03)		0.063	19	18	0.61 (0.25–1.46)	0.275
G/G	6	1	0.11 (0.01–0.97)		0.058	9	2	0.14 (0.03–0.74)*	0.016*
Natal sex within rs1818033 (n=187, crude analysis)									
Genotypes	Natal sex	Cis group		Trans group		OR (95% CI)		*P*-value	
C/C	Females	18		28		**Reference 1.00**		–	
	Males	16		27		1.08 (0.46–2.55)		0.870	
C/G	Females	26		18		**Reference 1.00**		–	
	Males	19		18		1.37 (0.57–3.30)		0.491	
G/G	Females	6		1		**Reference 1.00**		–	
	Males	9		2		1.33 (0.10–18.19)		0.840	
Test for interaction in the trend: 0.85									
rs1818033 within natal sex (n=187, crude analysis)									
Natal sex	Genotypes	Cis group		Trans group		OR (95% CI)		*P*-value	
Females	C/C	18		28		**Reference 1.00**		–	
	C/G	26		18		0.45 (0.19–1.03)		0.063	
	G/G	6		1		0.11 (0.01–0.97)		0.058	
Males	C/C	16		27		**Reference 1.00**		–	
	C/G	19		18		0.56 (0.23–1.37)		0.204	
	G/G	9		2		0.13 (0.03–0.69)		0.010*	
Test for interaction in the trend: 0.93									

* Reached statistical significance.

### 3.1 Haplotype analysis

We carried out the haplotype analysis of the two polymorphisms at the mGluR7 that passed the Bonferroni correction (D: 0.1215; D’: 0.9993; r: 0.6883 and *P*: 0). Linkage disequilibrium analysis between polymorphisms rs9838094 and rs1818033 showed that allele G from polymorphism rs9838094 was linked to allele C from polymorphism rs1818033 (haplotype 1: GC) (**Table 5**) and forms the most frequent haplotype (68.88%) in transgender and cisgender populations. Respect to the haplotype 2 (TG), it was more frequent in the cisgender population with an OR≤ 0.34 (0.19 - 0.62; P ≤ 0.0006; global haplotype association P ≤ 0.0004). Haplotype interaction analysis with the covariate sex assigned at birth showed that the haplotype T-G was more frequent in the cisgender population for both, the male, and the female populations, with no evidence of sex cross-interaction (**Table 5**).

**Table 5:** Haplotype and Haplotype interaction analyses with the covariate natal sex. The table shows the haplotype frequencies estimation and the haplotype association with GI.

Haplotype analysis						
Haplotype frequencies estimation (n=188)						
Haplotypes	rs9838094	rs1818033	Total	Cis group	Trans group	Cumulative frequency
**1**	G	C	0.6888	0.6011	0.7766	0.6888
**2**	T	G	0.1786	0.2447	0.1117	0.8674
**3**	G	G	0.1326	0.1543	0.1117	1
**4**	T	C	0	0	0	1
Haplotype association with response (n=188, adjusted by sex)						
	rs9838094	rs1818033	Freq	OR (95% CI)		*P*-value
**1**	G	C	0.6888	**Reference 1.00**		—
**2**	T	G	0.1781	0.34 (0.19 - 0.62)		6.00E-04*†
**3**	G	G	0.133	0.54 (0.28 - 1.04)		0.065
Global haplotype association p-value: 0.00046*†						
Haplotype interaction analysis with covariate natal sex						
Haplotype and sex cross-classification interaction table (n=188, crude analysis)						
Haplotype	Frequency	Females	*P*-value	Males		*P*-value
		OR (95% CI)		OR (95% CI)		
**GC**	0.6888	**Reference 1.00**	–	1.13 (0.49 - 2.58)		0.785
**GG**	0.1332	0.47 (0.19 - 1.19)	0.106	0.70 (0.26 - 1.90)		0.491
**TG**	0.178	0.34 (0.14 - 0.82)	0.016*	0.38 (0.15 - 0.95)		0.039*
Interaction p-value: 0.91						
Haplotypes within sex (n=188, crude analysis)						
**GC**	0.6888	**Reference 1.00**	–	**Reference 1.00**		**–**
**GG**	0.1332	0.47 (0.19 - 1.19)	0.106	0.62 (0.25 - 1.57)		0.312
**TG**	0.178	0.34 (0.14 - 0.82)	0.016*	0.34 (0.15 - 0.77)		0.009*
Sex within haplotypes (n=188, crude analysis)						
		OR (95% CI)		OR (95% CI)		
**GC**	0.6888	**Reference 1.00**	–	1.13 (0.49 - 2.58)		0.785
**GG**	0.1332	**Reference 1.00**	–	1.49 (0.48 - 4.62)		0.499
**TG**	0.178	**Reference 1.00**	–	1.11 (0.40 - 3.10)		0.852

* Reached statistical significance.

† Passed the Bonferroni correction.

## 4 Discussion

Seventy-four polymorphisms located at the metabotropic glutamate receptors (mGluRs) were analyzed at three levels of significance (*P ≤* 0.05, *P ≤* 0.005 and *P ≤* 0.0005), founding 27 polymorphisms that reached statistical significance, two of which, located at the mGluR7, passed the Bonferroni correction. This is the first communication on the involvement of mGluR7 in gender identity.

Today, it is widely accepted that estradiol can act independently of the classical nuclear receptors, ERα and ERβ, by activating membrane-localized receptors (mER). Recent research indicates that mER signaling through mGluRs is an important and rapid mechanism by which estrogens can modulate neuronal and glial physiology, affecting various aspects of nervous system function (21, 23). There are data showing interaction between mGluRs and the ERs in several brain regions (23) that show sex differences in animals (34) and humans (35, 36). Sexual differentiation of the brain is developed by the intertwining work of gonadal hormones and neurotransmitters (37, 38). There are brain structural (6) and functional (7) differences between male and female cisgender and transgender people and a neurodevelopmental theory was proposed to explain these differences (6). The polymorphisms analyzed here show that some SNPs in the mGluR7 and mGluR5 receptors are more prevalent in transgender than in cisgender populations, suggesting the involvement of these receptors, at least mGluR7, in the development of transgender identity.

It is becoming clear that ER signaling through mGluRs is one important and rapid mechanism by which estrogens can modulate neuron and glial physiology, ultimately impacting various aspects of nervous system function (23). Studies of ERα and ERβ overexpression showed that a subpopulation of these classical receptors is trafficked to the membrane (membrane-bound estrogen receptors, mERs) activating intracellular signaling (23, 39). In addition, mERs interact with metabotropic glutamate receptors (mGluRs) (40, 41), such that mER/mGluR coupling initiates G-protein signaling cascades that rapidly affect cellular excitability and gene expression (19), influencing neuronal physiology, structure and behavior (23, 42).

Estrogens are known to be potent regulators of neuronal structure (increasing dendrite length and spine density) (43). In addition, rapid effects of estrogens have also been described for sexual behavior (44). On the basis of the above, we believe that it would be also necessary to analyze polymorphisms located in the GPER-1 (G protein-coupled estrogen receptor 1) gene, a specific membrane estrogen receptor (45, 46) that have not yet been analyzed in the transgender population.

On the other hand, the great variety of receptor pairs that are possible due to the existence of multiple subtypes of ER (α and β) and mGluRs (1–8 and subtypes), gives rise to a great diversity of molecular results that affect processes as diverse as cognition, motivation, movement or sexual behavior. For example, the mGluR5 plays an important role in the regulation of synaptic plasticity and the modulation of the neural network activity (47, 48) And both, mGluR5 and mGluR7 influence rodent male sexual behavior (24–26).

mGluR5, which is mainly located at the postsynaptic regions (49), is an important regulator of both excitatory and inhibitory pathways, and alterations in its expression are often related to a number of neurological and psychiatric conditions, including epilepsy, anxiety, and autism spectrum disorder (ASD) (50).

mGluR7, which is expressed presynaptic (51), is also widely distributed in the brain (52), and couples to G proteins. Under high glutamate concentrations, mGluR7 acts as auto-receptor to inhibit further neurotransmitter release (53). Moreover, mGluR7 also functions as a hetero receptor inhibiting GABA release (51, 54). Thus, activation of these receptors modulates glutamate release (55, 56) controlling the excitatory synapse function (57).

In addition, evidence suggests that mGluR7 exhibits a basal signaling (constitutive activity), even in the absence of its natural ligand (glutamate). When expressed in neurons, mGluR7 shows detectable basal calcium channel modulation without the need for strong receptor activation. This constitutive activity implies that mGluR7 may have a physiological role even when not fully activated by glutamate (58).

On the other hand, mGluR5 and mGluR7 are known to mediate emotional and social behavior (57, 59, 60). Thus, some studies have shown that mGluR5 protein levels in the amygdala increase concomitantly with anxiety behaviors in adolescent mice after two weeks of isolation (61). mGluR7 is also implicated in the pathogenesis of depression. Thus, some genetic polymorphisms located in this receptor are known to increase susceptibility to depression (62). Given that in the present study all transgender individuals who participated showed GD, we believe that to better understand this trait in the future, it might help to address the study of different polymorphisms located in mGluR5–7 genes.

Moreover, the GABAergic/glutamatergic system has been implicated in suicidal behaviors (63) and transgender population shows higher suicide ideation and attempts (28, 29). Post mortem studies of the dorsal prefrontal cortex show a generalized disruption of the regulation of the glutamate receptors in suicidals (32). Taking all this together, we believe that more attention should be paid to the possible link between mGluRs polymorphisms and suicide risk in transgender people. But we should not underestimate the obvious social, cultural, relational, and multiple other difficulties experienced by people with a non-conforming transgender identity. Not being fully accepted in society is an obvious non-negligible risk factor for mood disorders and suicide in transgender populations.

## 5 Limitations and strengths

Our work has limitations as well as strengths. The main limitation is that the population analyzed was small. It would be necessary to replicate the data in a larger transgender population. Moreover, as with other studies, there is a lack of representativeness of cases because participants were recruited from a gender unit, this could be contributing to selection bias.

The strengths of our study are the control of the sample homogeneity. Although transgender people comprise a heterogeneous population, they can be stratified according to variables such as age of onset of dysphoria, geographic origin, and ethnicity. Our sample was rigorously controlled in all these aspects.

## Data availability statement

The original contributions presented in the study are included in the article/**Supplementary material**. Further inquiries can be directed to the corresponding author.

## Ethics statement

The studies involving humans were approved by Ethical committees of the National University of Distance Education (UNED, Madrid). The studies were conducted in accordance with the local legislation and institutional requirements. The participants provided their written informed consent to participate in this study.

## Author contributions

RF: Conceptualization, Data curation, Formal analysis, Funding acquisition, Investigation, Methodology, Project administration, Resources, Supervision, Validation, Visualization, Writing – original draft, Writing – review & editing. KR: Data curation, Formal analysis, Investigation, Writing – original draft, Writing – review & editing. RL: Formal analysis, Writing – original draft, Writing – review & editing, Data curation, Investigation. EG-G: Conceptualization, Investigation, Writing – original draft, Writing – review & editing. MM: Conceptualization, Investigation, Writing – original draft, Writing – review & editing. AG: Conceptualization, Investigation, Writing – original draft, Writing – review & editing, Funding acquisition, Methodology, Project administration, Resources, Supervision, Validation, Visualization. EP: Funding acquisition, Investigation, Methodology, Project administration, Supervision, Validation, Writing – original draft, Writing – review & editing, Resources, Visualization, Conceptualization, Data curation, Formal analysis.

## Supplementary material

Supplementary Table 1 Characteristics of the analyzed population.
