# Supplementary material for: Analysis of single nucleotide polymorphisms of the metabotropic glutamate receptors in a transgender population
Source: Front Endocrinol (Lausanne). 2024 Jun 11;15:1382861. doi: 10.3389/fendo.2024.1382861 (PMC11196815; doi:10.3389/fendo.2024.1382861)
Supplement: Supplementary Table 1 — Characteristics of the analyzed population. [file DataSheet_1.pdf]

Supplemental Table 1

ID	Group	Age first visit	Place of birth	Ethnicity	First symptoms
TW_038	TW	35.01	Spain	Caucasian	Early onset
TW_040	TW	40.04	Spain	Caucasian	Early onset
TW_044	TW	20.02	Spain	Caucasian	Early onset
TW_055	TW	30.11	Spain	Caucasian	Early onset
TW_056	TW	19.04	Spain	Caucasian	Early onset
TW_057	TW	18.07	Spain	Caucasian	--
TW_058	TW	29.09	Spain	Caucasian	Early onset
TW_061	TW	23.03	Spain	Caucasian	Early onset
TW_062	TW	46.04	Spain	Caucasian	--
TW_067	TW	45.07	Spain	Caucasian	--
TW_078	TW	36.10	Spain	Caucasian	Early onset
TW_079	TW	34.06	Spain	Caucasian	Early onset
TW_081	TW	30.02	Spain	Caucasian	Early onset
TW_086	TW	27.02	Spain	Caucasian	Early onset
TW_091	TW	25.11	Spain	Caucasian	Early onset
TW_094	TW	34.04	Spain	Caucasian	Early onset
TW_096	TW	40.07	Spain	Caucasian	Early onset
TW_097	TW	38.02	Spain	Caucasian	Early onset
TW_099	TW	36.05	Spain	Caucasian	Early onset
TW_101	TW	46.01	Spain	Caucasian	Early onset
TW_102	TW	20.12	Spain	Caucasian	Early onset
TW_103	TW	52.08	Spain	Caucasian	Early onset
TW_104	TW	27.01	Spain	Caucasian	Early onset
TW_105	TW	29.03	Spain	Caucasian	Early onset
TW_106	TW	38.10	Spain	Caucasian	Early onset
TW_113	TW	32.12	Spain	Caucasian	--
TW_116	TW	35.03	Spain	Caucasian	Early onset
TW_118	TW	23.05	Spain	Caucasian	Early onset
TW_119	TW	35.01	Spain	Caucasian	Early onset
TW_122	TW	26.07	Spain	Caucasian	Early onset
TW_126	TW	20.11	Spain	Caucasian	Early onset
TW_127	TW	20.03	Spain	Caucasian	Early onset
TW_128	TW	31.10	Spain	Caucasian	--
TW_129	TW	40.04	Spain	Caucasian	Early onset
TW_130	TW	32.09	Spain	Caucasian	Late onset
TW_165	TW	18.05	Spain	Caucasian	--
TW_166	TW	22.05	Spain	Caucasian	Early onset
TW_169	TW	27.10	Spain	Caucasian	--
TW_172	TW	40.11	Spain	Caucasian	Early onset
TW_173	TW	20.04	Spain	Caucasian	--
TW_185	TW	29.02	Spain	Caucasian	Early onset
TW_190	TW	47.01	Spain	Caucasian	Early onset
TW_200	TW	38.09	Spain	Caucasian	Early onset

TW_207	TW	25.07	Spain	Caucasian	Early onset
TW_227	TW	18.06	Spain	Caucasian	Early onset
TW_232	TW	31.02	Spain	Caucasian	Early onset
TW_236	TW	26.01	Spain	Caucasian	Early onset
TM_030	TM	26.09	Spain	Caucasian	Early onset
TM_041	TM	18.05	Spain	Caucasian	Early onset
TM_043	TM	20.06	Spain	Caucasian	Early onset
TM_045	TM	42.01	Spain	Caucasian	Early onset
TM_046	TM	34.05	Spain	Caucasian	Early onset
TM_051	TM	24.07	Spain	Caucasian	Early onset
TM_063	TM	30.12	Spain	Caucasian	Early onset
TM_064	TM	20.01	Spain	Caucasian	Early onset
TM_069	TM	19.07	Spain	Caucasian	Early onset
TM_071	TM	27.03	Spain	Caucasian	Early onset
TM_073	TM	40.09	Spain	Caucasian	Early onset
TM_082	TM	21.01	Spain	Caucasian	Early onset
TM_088	TM	24.07	Spain	Caucasian	Early onset
TM_092	TM	21.09	Spain	Caucasian	Early onset
TM_093	TM	24.03	Spain	Caucasian	Early onset
TM_107	TM	20.05	Spain	Caucasian	Early onset
TM_110	TM	32.11	Spain	Caucasian	--
TM_111	TM	18.04	Spain	Caucasian	--
TM_112	TM	24.09	Spain	Caucasian	--
TM_115	TM	27.12	Spain	Caucasian	--
TM_120	TM	21.10	Spain	Caucasian	Early onset
TM_121	TM	36.02	Spain	Caucasian	Early onset
TM_136	TM	23.01	Spain	Caucasian	Early onset
TM_140	TM	23.05	Spain	Caucasian	Early onset
TM_141	TM	22.01	Spain	Caucasian	Early onset
TM_142	TM	27.11	Spain	Caucasian	Early onset
TM_143	TM	26.08	Spain	Caucasian	Early onset
TM_144	TM	41.04	Spain	Caucasian	Early onset
TM_146	TM	27.02	Spain	Caucasian	Early onset
TM_156	TM	25.06	Spain	Caucasian	Early onset
TM_163	TM	30.10	Spain	Caucasian	Early onset
TM_171	TM	28.08	Spain	Caucasian	Early onset
TM_175	TM	31.05	Spain	Caucasian	Early onset
TM_176	TM	21.02	Spain	Caucasian	Early onset
TM_177	TM	22.10	Spain	Caucasian	Early onset
TM_179	TM	23.11	Spain	Caucasian	Early onset
TM_180	TM	36.03	Spain	Caucasian	Early onset
TM_182	TM	25.01	Spain	Caucasian	Early onset
TM_187	TM	31.05	Spain	Caucasian	Early onset
TM_194	TM	31.09	Spain	Caucasian	Early onset
TM_197	TM	48.12	Spain	Caucasian	Early onset
TM_209	TM	23.05	Spain	Caucasian	Early onset

TM_211	TM	22.09	Spain	Caucasian	Early onset
TM_216	TM	21.07	Spain	Caucasian	Early onset
TM_217	TM	20.04	Spain	Caucasian	Early onset
TM_239	TM	22.10	Spain	Caucasian	Early onset
TM_250	TM	18.03	Spain	Caucasian	Early onset
CM_1_19	CM	35.12	Spain	Caucasian	--
CM_10_19	CM	40.07	Spain	Caucasian	--
CM_12_19	CM	20.01	Spain	Caucasian	--
CM_13_19	CM	30.07	Spain	Caucasian	--
CM_14_19	CM	19.03	Spain	Caucasian	--
CM_15_19	CM	18.07	Spain	Caucasian	--
CM_17_19	CM	29.05	Spain	Caucasian	--
CM_18_19	CM	23.09	Spain	Caucasian	--
CM_19_19	CM	48.02	Spain	Caucasian	--
CM_2_19	CM	45.04	Spain	Caucasian	--
CM_20_19	CM	36.12	Spain	Caucasian	--
CM_21_19	CM	34.09	Spain	Caucasian	--
CM_22_19	CM	30.05	Spain	Caucasian	--
CM_23_19	CM	27.07	Spain	Caucasian	--
CM_24_19	CM	25.01	Spain	Caucasian	--
CM_26_19	CM	34.04	Spain	Caucasian	--
CM_27_19	CM	40.09	Spain	Caucasian	--
CM_28_19	CM	38.01	Spain	Caucasian	--
CM_29_19	CM	36.09	Spain	Caucasian	--
CM_3_19	CM	46.10	Spain	Caucasian	--
CM_30_19	CM	20.09	Spain	Caucasian	--
CM_31_19	CM	52.03	Spain	Caucasian	--
CM_32_19	CM	32.09	Spain	Caucasian	--
CM_33_19	CM	29.01	Spain	Caucasian	--
CM_34_19	CM	38.06	Spain	Caucasian	--
CM_35_19	CM	32.01	Spain	Caucasian	--
CM_36_19	CM	35.10	Spain	Caucasian	--
CM_37_19	CM	23.04	Spain	Caucasian	--
CM_38_19	CM	35.09	Spain	Caucasian	--
CM_39_19	CM	26.05	Spain	Caucasian	--
CM_4_19	CM	21.03	Spain	Caucasian	--
CM_40_19	CM	20.12	Spain	Caucasian	--
CM_41_19	CM	31.10	Spain	Caucasian	--
CM_5_19	CM	40.04	Spain	Caucasian	--
CM_52_19	CM	38.08	Spain	Caucasian	--
CM_54_19	CM	18.04	Spain	Caucasian	--
CM_55_19	CM	22.07	Spain	Caucasian	--
CM_56_19	CM	27.04	Spain	Caucasian	--
CM_57_19	CM	43.01	Spain	Caucasian	--
CM_58_19	CM	20.06	Spain	Caucasian	--
CM_59_19	CM	29.08	Spain	Caucasian	--

CM_60_19	CM	47.11	Spain	Caucasian	--
CM_7_19	CM	38.06	Spain	Caucasian	--
CM_9_19	CM	25.12	Spain	Caucasian	--
CW_1_19	CW	18.07	Spain	Caucasian	--
CW_10_19	CW	31.11	Spain	Caucasian	--
CW_12_19	CW	26.07	Spain	Caucasian	--
CW_13_19	CW	26.04	Spain	Caucasian	--
CW_14_19	CW	18.12	Spain	Caucasian	--
CW_15_19	CW	20.07	Spain	Caucasian	--
CW_17_19	CW	35.03	Spain	Caucasian	--
CW_18_19	CW	34.06	Spain	Caucasian	--
CW_19_19	CW	24.07	Spain	Caucasian	--
CW_2_19	CW	30.02	Spain	Caucasian	--
CW_20_19	CW	20.09	Spain	Caucasian	--
CW_21_19	CW	19.10	Spain	Caucasian	--
CW_23_19	CW	27.01	Spain	Caucasian	--
CW_24_19	CW	40.04	Spain	Caucasian	--
CW_25_19	CW	21.07	Spain	Caucasian	--
CW_27_19	CW	24.01	Spain	Caucasian	--
CW_28_19	CW	20.02	Spain	Caucasian	--
CW_29_19	CW	24.05	Spain	Caucasian	--
CW_3_19	CW	20.04	Spain	Caucasian	--
CW_30_19	CW	30.01	Spain	Caucasian	--
CW_31_19	CW	24.10	Spain	Caucasian	--
CW_32_19	CW	30.09	Spain	Caucasian	--
CW_33_19	CW	35.03	Spain	Caucasian	--
CW_34_19	CW	21.01	Spain	Caucasian	--
CW_35_19	CW	36.05	Spain	Caucasian	--
CW_36_19	CW	23.12	Spain	Caucasian	--
CW_37_19	CW	23.06	Spain	Caucasian	--
CW_38_19	CW	22.02	Spain	Caucasian	--
CW_39_19	CW	27.01	Spain	Caucasian	--
CW_4_19	CW	26.12	Spain	Caucasian	--
CW_40_19	CW	22.06	Spain	Caucasian	--
CW_41_19	CW	27.11	Spain	Caucasian	--
CW_5_19	CW	25.03	Spain	Caucasian	--
CW_52_19	CW	30.09	Spain	Caucasian	--
CW_53_19	CW	28.03	Spain	Caucasian	--
CW_55_19	CW	31.12	Spain	Caucasian	--
CW_56_19	CW	21.04	Spain	Caucasian	--
CW_57_19	CW	22.08	Spain	Caucasian	--
CW_58_19	CW	23.07	Spain	Caucasian	--
CW_60_19	CW	36.01	Spain	Caucasian	--
CW_61_19	CW	25.10	Spain	Caucasian	--
CW_62_19	CW	25.04	Spain	Caucasian	--
CW_63_19	CW	31.12	Spain	Caucasian	--

CW_64_19	CW	23.09	Spain	Caucasian	--
CW_65_19	CW	23.08	Spain	Caucasian	--
CW_66_19	CW	22.01	Spain	Caucasian	--
CW_67_19	CW	21.05	Spain	Caucasian	--
CW_68_19	CW	20.06	Spain	Caucasian	--
CW_69_19	CW	22.11	Spain	Caucasian	--
CW_9_19	CW	22.04	Spain	Caucasian	--
