# Supplementary material for: Analysis of single nucleotide polymorphisms of the metabotropic glutamate receptors in a transgender population
Source: Front Endocrinol (Lausanne). 2024 Jun 11;15:1382861. doi: 10.3389/fendo.2024.1382861 (PMC11196815; doi:10.3389/fendo.2024.1382861)
Supplement: Supplementary file 2 [file DataSheet_2.pdf]

**Supplemental Table 2**

Name of the polymorphisms analyzed (SNP-ID), chromosome localization and localization at the metabotropic glutamate receptors. * Polymorphism with significant differences at level $P \leq 0.05$. ** Polymorphism with significant differences at level $P \leq 0.005$. *** Polymorphism with significant differences at level $P \leq 0.0005$

| Metabotropic glutamate receptor name | Chromosome localization | SNP-ID |
|---|---|---|
| mGluR1 | 6q24.3 | rs77376872* |
| | | rs2256998 |
| | | rs2247666 |
| mGluR3 | 7q21.11-q21.12 | rs144397319 |
| mGluR4 | 6p21.31 | rs10947475 |
| | | rs6904593* |
| | | rs10947476 |
| | | rs9380405 |
| mGluR5 | 11q14.2-q14.3 | rs507187* |
| | | rs603472 |
| | | rs12364488* |
| | | rs7924925* |
| | | rs1042602 |
| mGluR7 | 3p26.1 | rs9872989* |
| | | rs62237166* |
| | | rs7633040* |
| | | rs73013949 |
| | | rs1876615* |
| | | rs62237207** |
| | | rs1396402 |
| | | rs62237212** |
| | | rs13080594 |
| | | rs62237216** |
| | | rs73809085* |
| | | rs62237226** |
| | | rs1603876 |

| |
|---|
| rs579837 |
| rs712776* |
| rs12634429* |
| Affx-22760456 |
| **rs9838094***** |
| rs371097 |
| **rs1818033***** |
| rs35154901 |
| rs1516302* |
| rs9865821 |
| rs1396409* |
| rs11708019 |
| rs4095095* |
| rs6775292 |
| rs79706002 |
| rs13071462 |
| rs1901046 |
| rs7623046 |
| rs13068639 |
| rs9860274 |
| rs56002938* |
| rs9850541 |
| rs36069901 |
| rs1450100 |
| rs3804945* |
| rs17752444 |
| rs55719233 |
| rs6443099 |
| rs12491287 |
| rs1508717 |
| rs9879939 |
| rs79706002 |
| rs2229902 |

| | | |
|---|---|---|
| | | rs28627576 |
| mGluR8 | 7q31.33 | rs1419441 |
| | | rs2097617 |
| | | rs11762115* |
| | | rs1419391 |
| | | rs7782149** |
| | | rs17867159 |
| | | rs75846534 |
| | | rs9640842 |
| | | rs4141415 |
| | | rs10269701 |
| | | Affx-29672839 |
| | | rs1557643 |
| | | rs2027952 |
| | | rs712701 |

This is a provisional file, not the final typeset article
